# Nuclear Medicine Training: Skills and Competencies Required for Practice in the 21st Century

**DOI:** 10.1055/s-0043-1769588

**Published:** 2023-05-22

**Authors:** Ismaheel O. Lawal

**Affiliations:** 1 Department of Radiology and Imaging Sciences, Emory University, Atlanta, Georgia, United States; 2 Department of Nuclear Medicine, University of Pretoria, Pretoria, South Africa

Nuclear medicine (NM) utilizes unsealed radiation sources to diagnose and treat diseases. In the NM team, the NM physician works in collaboration with many other NM professionals who play critical roles in care delivery to patients. The NM team, therefore, consists of the NM physicians, the radiochemists, the medical physicists, and NM technologists, and others. Each of these groups in the NM team has made significant contributions to the field of NM, resulting in amazing growth over the last two decades or so. 1 This growth with implications for a promising feature for NM has been in the form of improvement in instrumentation, advances in radiopharmaceutical synthesis, the introduction of novel diagnostic and therapeutic radiopharmaceuticals, optimization of dosimetry methods, and, consequently, broadening of the applications of NM techniques in the clinics. While this growth occurring in all aspects of the field has made the future of the profession exciting, it has also come with a need for residency training to evolve to produce NM physicians with the requisite skill sets and competencies that make them suitable to deliver efficient care in the 21st century. In this editorial, I will focus on the emerging skill sets and competencies that NM trainees need to acquire in their residency training to render fit-for-purpose diagnostic and therapeutic NM care in the 21st century.

The introduction of positron emission tomography (PET) cameras to the clinics in the 2000s marked a turning point in the fortune of NM practice. Fluorine-18 fluorodeoxyglucose (FDG), a marker of glucose metabolism, has been the workhorse of PET imaging and utilized for several indications across different oncologic and nonmalignant conditions. Two decades of clinical use of FDG have led to the identification of many of its limitations, resulting in the development of PET radiopharmaceuticals approved for use in routine patient care. These novel radiopharmaceuticals include ^68^ Ga-DOTATATE for neuroendocrine imaging and ^68^ Ga- or ^18^ F-labeled prostate-specific membrane antigen (PSMA) for prostate cancer imaging. Other novel tracers with great promise for use in routine clinical practice include radioligand targeting fibroblast activation protein expressed by cancer-associated fibroblast and CXCR4, a chemokine expressed in different inflammatory and malignant conditions. Advances in camera design have occurred in parallel with the development of novel radiopharmaceuticals. Hybrid PET/CT cameras have replaced the initial stand-alone PET camera, and more recently, PET/MR is getting a broadening clinical utilization. The total-body PET camera brings about many new possibilities previously unimaginable. Overall, improving camera design and electronics has led to better camera spatial resolution and sensitivity.

NM imaging is a functional imaging that requires a thorough understanding of the pathophysiological perturbation in diseases. The need for NM practitioners to acquire competencies for morphologic imaging interpretation arose when hybrid PET imaging became the norm in the clinics. This need for this skill set was identified by NM training bodies in different countries leading to various forms of modifications to NM training, inculcating variable lengths of training in radiology in the NM training curriculum. In the United States, about three pathways exist for NM trainees to obtain some radiology exposure to acquire competencies in reading morphologic imaging. 2 3 In the United Kingdom, a 6-year training program consisting of sequential 3-year radiology and 3-year NM training has been recently introduced to address this need. 4 Most recently, the Dutch made, perhaps, the boldest attempt at addressing the need for radiology exposure for NM trainees by integrating the NM and radiology training. 5 Several other training bodies have made modifications to address this need in different countries. 6 While changes made so far have got their strengths by broadening the knowledge base of NM physicians entering specialist practice, none of them is without some drawbacks. Therefore, further refinement is needed in the modifications made to NM training in different jurisdictions. Future iterations in modifications to training should take cognizant of some factors that may endanger the profession rather than strengthen it. The training duration should not be unduly lengthened. It is difficult to justify a NM training duration longer than radiology training, as the scope of radiology, with its many modalities, is broader than that of NM. Modifying NM and radiology training must be done cautiously to avoid the recently reported Dutch experience where trainees are diverting more toward radiology subspecialties than toward NM. 7

Continuing improvement in camera performance is increasing lesion detection rate with an attendant risk of scan over-reading. Lesions with subtle avidity for tracer that would be barely discernible above the surrounding background activity on older PET systems are now easily detectable on newer PET systems with much-improved camera performance. This indeed augurs well for lesion detection. The drawback to this improvement in lesion detectability is the increasing detection of radiotracer-avid benign lesions with an attendant increase in false positivity rate. A good understanding of radiology may help separate benign and inconsequential lesions from lesions of interest being evaluated in a given disease. This point demonstrates that the need for radiological skillsets among NM practitioners is getting more important as the field advances. Overall, a good knowledge of morphologic imaging improves the competency of an NM practitioner in reading NM scans.

Pundits are unanimous in submitting that radionuclide therapy will be the primary driver of the current and future success of NM. NM is at the forefront of personalized medicine, leveraging on the use of theranostics by selecting only patients who express a target of interest to selectively deliver a lethal radiation dose to the tumor while sparing normal surrounding tissues. Theranostics has been applied for decades in NM through the application of radioiodine for imaging and therapy of thyroid disorders. Recent major breakthroughs are the development and approval of ^68^ Ga/ ^177^ Lu-DOTATATE and ^68^ Ga/ ^177^ Lu-PSMA as theranostic pairs for managing differentiated neuroendocrine tumors and metastatic castration-resistant prostate cancer (mCRPC), respectively. 8 9 More theranostic pairs are in the developmental pipelines. Radiun-223 dichloride leverages on the energetic α particle for the effective treatment of patients with bone-predominant mCRPC. 10 Novel α-emitting radiopharmaceuticals are now being investigated for treating mCRPC, neuroendocrine tumors, and other malignancies. 11 12

To guarantee the future success of theranostics in NM and the profession as a whole, new competencies must be developed among NM practitioners to efficiently care for patients. While therapy is not new to NM, prior therapies such as radioiodine treatment and radiosynovectomy were administered to less sick patients. The recently introduced theranostic agents are administered in much sicker patients, such as mCRPC patients with much more complex health needs. So, our patient-facing skills need to evolve to meet the increasing demand required by the patients we will be providing care to in the 21st century. These skills should cover areas including knowing the alternatives to the treatment we offer, counseling patients and taking informed consent, assessing patients to determine their suitability and fitness for treatment, administering radionuclide therapy, reviewing laboratory results, and evaluating patients for treatment-induced toxicities, following up patients for treatment response assessment, and others. 13 The decision to administer radionuclide therapy should ideally be reached in a multidisciplinary setting with the patient in the driver seat. 14 The model of post-treatment care to patients following radionuclide therapy (such as managing treatment-induced toxicities) will vary in different jurisdictions, but in most instances, it will be done in cooperation between the NM team and the oncologists.

Before now, as imagers, NM physicians perform scan readouts while the managing team, in most instances, reviews the scan findings with the patients. As NM physicians become more involved with delivering care to patients due to the broadening of the applications of theranostics, a need for the NM physicians to be competent in discussing scan findings with patients, which sometimes involves breaking bad news, becomes imperative. This signifies the changing role of the NM physician from more predominantly an imager role to an all-rounder physician with imaging and therapy competencies. 15 Response assessment after treatment is still largely done using morphologic imaging response criteria such as the response assessment criteria in solid tumors (RESIST criteria). To do this effectively, the NM physicians need competencies in interpreting morphologic imaging, highlighting another importance for NM trainees to acquire radiology skills during their training.

In conclusion, NM is experiencing seismic growth with a promisingly bright future for the profession. The growth trajectory demands retooling the skill toolbox of the NM physician. It also calls for modifications to the NM training curriculum so that new professionals can provide competent patient care. The new skill sets must address new demands for diagnostic and therapeutic NM practice in the 21st century. Many NM training bodies have risen to these challenges by introducing changes to NM training in their jurisdictions. These changes have been largely successful in most instances, though unintended negative consequences have been experienced as well. Future attempts at addressing this important need for our profession must leverage on the success of previous exercises while avoiding the unintended negative consequences identified. The critical role that retooling and reskilling of NM practitioners play in advancing the success of NM practice is experiencing demands that conversations on this subject continue so that we can create dynamic training programs that address the needs for the delivery of competent care as the profession evolves.

## References

[1] Vaz S C Oliveira F Herrmann K Veit-Haibach P Nuclear medicine and molecular imaging advances in the 21st century Br J Radiol 2020 93(1110):2.0200095E7 10.1259/bjr.20200095 PMC10993229 32401541

[2] Harolds J A Oates M E Guiberteau M J Ghesani M Scanlon M H Iagaru A H New training pathways to dual certification in nuclear medicine and radiology J Nucl Med 2015 56 06 17N 18N 26033625

[3] Segall G M Grady E E Fair J R Ghesani M V Gordon L Nuclear medicine training in the United States J Nucl Med 2017 58 11 1733 1734 28986514 10.2967/jnumed.117.200857

[4] Neilly B Dizdarevic S Prvulovich L Buscombe J Lewington V Nuclear medicine training and practice in the UK Eur J Nucl Med Mol Imaging 2016 43 04 800 803 26592940 10.1007/s00259-015-3255-7

[5] Velleman T Kwee T C Dierckx R AJO Ongena Y P Noordzij W The integrated nuclear medicine and radiology residency program in the Netherlands: strengths and potential areas for improvement according to nuclear medicine physicians and radiologists Eur J Nucl Med Mol Imaging 2022 49 09 3016 3022 35194672 10.1007/s00259-022-05699-8 PMC9250465

[6] Muylle K Maffioli L Nuclear medicine training in Europe: “all for one, one for all” J Nucl Med 2017 58 12 1904 1905 29051344 10.2967/jnumed.117.201012

[7] Johannes C Ken H The disappearing act of nuclear medicine in the Netherlands: just a new trick by the great Harry Houdini? J Nucl Med 2021 62 07 903 904 33712538 10.2967/jnumed.121.262190

[8] NETTER-1 Trial Investigators Strosberg J El-Haddad G Wolin E Phase 3 trial of ^177^ Lu-dotatate for midgut neuroendocrine tumors N Engl J Med 2017 376 02 125 135 28076709 10.1056/NEJMoa1607427 PMC5895095

[9] VISION Investigators Sartor O de Bono J Chi K N Lutetium-177-PSMA-617 for metastatic castration-resistant prostate cancer N Engl J Med 2021 385 12 1091 1103 34161051 10.1056/NEJMoa2107322 PMC8446332

[10] ALSYMPCA Investigators Parker C Nilsson S Heinrich D Alpha emitter radium-223 and survival in metastatic prostate cancer N Engl J Med 2013 369 03 213 223 23863050 10.1056/NEJMoa1213755

[11] Sathekge M M Bruchertseifer F Vorster M Morgenstern A Lawal I O Global experience with PSMA-based alpha therapy in prostate cancer Eur J Nucl Med Mol Imaging 2021 49 01 30 46 34173838 10.1007/s00259-021-05434-9 PMC8712297

[12] Feuerecker B Kratochwil C Ahmadzadehfar H Morgenstern A Eiber M Herrmann K 2023 10.2967/jnumed.122.265353 37055224

[13] Lee S T Emmett L M Pattison D A The importance of training, accreditation, and guidelines for the practice of theranostics: the Australian perspective J Nucl Med 2022 63 06 819 822 35393349 10.2967/jnumed.122.263996

[14] Farolfi A Lima G M Oyen W Fanti S Molecular imaging and theranostics-a multidisciplinary approach Semin Nucl Med 2019 49 04 247 254 31227048 10.1053/j.semnuclmed.2019.02.002

[15] Bodei L Chiti A Modlin I M Scott A M Schöder H The path to the future: education of nuclear medicine therapeutic specialists as responsible physicians J Nucl Med 2019 60 12 1663 1664 31732675 10.2967/jnumed.119.232454

